# Effects of Synaptic Plasticity on Phase and Period Locking in a Network of Two Oscillatory Neurons

**DOI:** 10.1186/2190-8567-4-8

**Published:** 2014-04-29

**Authors:** Zeynep Akcay, Amitabha Bose, Farzan Nadim

**Affiliations:** 1 Department of Mathematical Sciences, New Jersey Institute of Technology, Newark, NJ, 07102, USA; 2 Federated Department of Biological Sciences, New Jersey Institute of Technology and Rutgers University, Newark, NJ, 07102, USA

**Keywords:** Phase locking, Oscillatory neural network, Phase response curve, Short-term synaptic plasticity

## Abstract

We study the effects of synaptic plasticity on the determination of firing period and relative phases in a network of two oscillatory neurons coupled with reciprocal inhibition. We combine the phase response curves of the neurons with the short-term synaptic plasticity properties of the synapses to define Poincaré maps for the activity of an oscillatory network. Fixed points of these maps correspond to the phase-locked modes of the network. These maps allow us to analyze the dependence of the resulting network activity on the properties of network components. Using a combination of analysis and simulations, we show how various parameters of the model affect the existence and stability of phase-locked solutions. We find conditions on the synaptic plasticity profiles and the phase response curves of the neurons for the network to be able to maintain a constant firing period, while varying the phase of locking between the neurons or vice versa. A generalization to cobwebbing for two-dimensional maps is also discussed.

## 1 Introduction

The output of a neuronal network, determined in part by the relative spiking times of its individual neurons, depends on the coordinated activity of its neurons. Observed phase relationships result from the combined effects of individual cells and synaptic connections whose properties change dynamically. For example, individual neurons in a network can differ in their intrinsic properties, being silent, spiking or bursting; different neurons can have different responses to the synaptic inputs they receive, and the synaptic inputs themselves can differ widely. These different characteristics all play a role in determining the resulting network activity. Determining how these dynamically varying components work together to influence the network activity is a question of considerable interest.

Many studies have explored the question of how period and phase are determined in an oscillatory neuronal network [1-7]. One of the main tools used in these studies is the phase response (or resetting) curve (PRC) of an individual neuron. The PRC measures how the phase of firing of an oscillatory neuron changes as a function of perturbations that it receives at different phases of its oscillation. In prior work [8], the PRC has been used to define a 1D map that measures the degree of network synchrony. This map allows for the analysis of the network activity in a reduced system by considering only the effect of the synaptic inputs on cycle length, rather than considering multiple dynamic variables. Several studies used similar methods to study the activity of neuronal networks [2,8-12]. PRC-based maps were also used to incorporate some properties of neurons or synapses. This approach was applied to understand synchronization of adapting neurons [2,5] as well as the effect of conduction delays on network synchrony [1,13]. 

In the current study, we are interested in predicting phase-locking by deriving maps that combine PRCs with information arising directly from synapses that display frequency-dependent short-term plasticity. Increases in presynaptic firing frequency can strengthen (facilitation) or weaken (depression) a synapse [14]. Some synapses show a combination of both, in which case the maximal synaptic amplitude is achieved at a specific presynaptic frequency [15] referred to as the preferred frequency of the synapse. Synaptic plasticity can be described with models having two variables, one for depression and the other for facilitation [15,16]. 

The main advance in our work is the derivation of tools for analyzing higher-dimensional maps that incorporate the effects of synaptic plasticity and provide predictions on circumstances under which an oscillatory network of neurons will phase-lock and at what period. In particular, we consider a network of two neurons, mutually coupled by inhibition in which the synaptic strength is frequency dependent. In deriving these maps, we must not only track the phases of each cell, but also the strength of each synapse. As a result, the 1D map that sufficed in prior studies needs to be replaced with 2D or 3D maps. For 2D maps, we derive a geometric method that generalizes the idea of cobwebbing. Namely, we show how iterations of the map can be tracked through different 2D surfaces. Moreover, projections of these surfaces onto a common plane yields two curves whose intersection is a fixed point of the map that corresponds to a phase-locked solution. We derive conditions on the PRCs and the parameters that govern synaptic plasticity of the neurons to show how a network can have a range of parameters over which the network period remains constant, but the phase of locking between cells changes, or vice versa. We also show that the methods derived apply to networks that are heterogeneous either in the intrinsic properties of individual cells, in their synapses, or both.

## 2 Model and Methods

### 2.1 Dynamics of Neurons

We use Morris–Lecar (M–L) model neurons to conduct our analysis [17]. An isolated M–L neuron is modeled by leak (*L*), potassium (*K*) and calcium (*Ca*) current. The *K* current is driven by a dynamic activation variable *w*, while the *Ca* current depends on an instantaneous function $m_{\infty }$ of the membrane voltage (*V*). When the neuron is synaptically coupled to another neuron, the synaptic current received from the other cell is also included in the equation governing the membrane voltage. For two M–L neurons coupled with synaptic inhibition, the equations for voltage *V* and *K* activation variable *w* are given by

(2.1) $$\begin{array}{rl} C\frac{dV_{i}}{dt}= & I_{\mathrm{app}}-({\bar{g}}_{L}(V_{i}-E_{L})+{\bar{g}}_{K}w(V_{i}-E_{K})+{\bar{g}}_{\mathrm{Ca}}m_{\infty }(V_{i})(V_{i}-E_{\mathrm{Ca}})\\ & +g_{j\to i}H(V_{j}-V_{\mathrm{th}})\cdot (V_{i}-E_{\mathrm{syn}}))\\ \frac{dw_{i}}{dt}= & \frac{w_{\infty }(V_{i})-w_{i}}{\tau_{w}(V_{i})} \end{array}$$

for i,j=1,2, i≠j, where $m_{\infty }(V)=0.5(1+\tanh((V-V_{a})/V_{b}))$, $w_{\infty }(V)=0.5(1+\tanh((V-V_{c})/V_{d}))$ and $\tau_{w}(V)=1/(\phi \cosh((V-V_{c})/2V_{d}))$. The conductances (in nS) are ${\bar{g}}_{L}=2$, ${\bar{g}}_{K}=8$, ${\bar{g}}_{\mathrm{Ca}}=4$, the reversal potentials (in mV) are $E_{L}=-60$, $E_{K}=-84$, $E_{\mathrm{Ca}}=120$ for the leak, potassium and calcium currents, respectively. The synaptic reversal potential $E_{\mathrm{syn}}$ is −80 mV, modeling an inhibitory synapse. Due to the presence of the Heaviside function $H(V-V_{\mathrm{th}})$, the synapses are all-or-none and activate (deactivate) instantaneously when the presynaptic voltage is above (below) the synaptic threshold $V_{\mathrm{th}}=0\text{ mV}$. The results described in this study remain qualitatively similar if the value of $V_{\mathrm{th}}$ is changed.

Below we will provide more details of the synaptic conductance $g_{\mathrm{pre}\to \mathrm{post}}$. We change the applied current $I_{\mathrm{app}}$ (in pA) between 41.2 and 44.9 to obtain a set of intrinsic periods (in ms) ranging between 100.3 and 180.83. The rest of the model parameters are C=20 pF, ϕ=0.067 (dimensionless), and $V_{a}=-1.2$, $V_{b}=18$, $V_{c}=12$, $V_{d}=17.4$ in mV. Throughout the paper, units for time are in msec.

### 2.2 Phase Response Curves

**Fig. 1:**
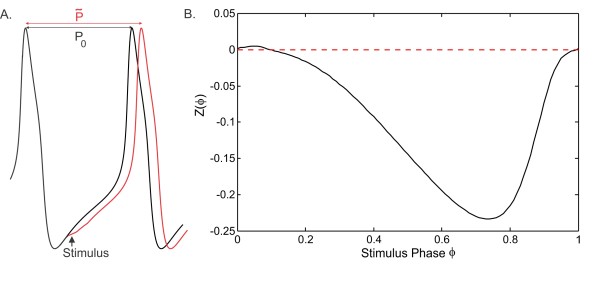
PRC due to synaptic input. **a** A brief perturbing current pulse stimulus (*arrow*) is used to measure the PRC as described in Eq. (2.2). **b** The PRC obtained from the Morris–Lecar model (2.1) neurons by inhibitory synaptic input. The parameters are $I_{\mathrm{app}}=42.2$, synaptic conductance $g_{\mathrm{pre}\to \mathrm{post}}=0.1$ and synaptic duration $t_{a}=14.3$

The phase response curve (PRC) of an oscillator describes how the time of the next spike of an oscillator changes depending on the phase at which it receives a perturbation (Fig. 1a). In general, the PRC can be computed numerically (for model neurons) or experimentally (for biological neurons) by injecting a brief perturbing current (such as a small current pulse) and measuring the effect of this perturbation on the cycle length as a function of the phase of the perturbing input. If the perturbation is infinitesimally small, then an infinitesimal phase response curve (iPRC) of the model neuron can be obtained by linearizing the governing differential equations about the limit cycle and solving the adjoint equation. In this work, we will use the term PRC to refer to responses calculated by direct perturbations, for example ones that imitate synaptic inputs. 

Denote by $P_{0}$ the intrinsic period of a cell. Suppose a perturbation is given at time *dt* after the firing of the cell. This yields a phase $\phi =dt/P_{0}$ of the perturbation. Denote by $\tilde{P}$ the time between when a cell fires prior to a perturbation and the subsequent firing of the cell when a perturbation is given at phase *ϕ*. Then we define the PRC as

(2.2) $$Z(\phi )=\frac{P_{0}-\tilde{P}}{P_{0}}$$

We have chosen parameters so that in the M–L model oscillations arise through a saddle node on invariant circle (SNIC) bifurcation. Neurons that oscillate through a SNIC bifurcation have a Type 1 iPRC [18], which is always of one sign. In the case of an inhibitory perturbation received by the neuron, the Type 1 iPRC is never positive and the next firing time is therefore delayed. A PRC obtained from our model neurons for a specific synaptic strength is shown in Fig. 1. It is computed by applying a perturbation of the form

$$I_{\mathrm{syn}}=g_{\mathrm{pre}\to \mathrm{post}}H(V_{\mathrm{pre}}-V_{\mathrm{th}})(V_{\mathrm{post}}-E_{\mathrm{syn}})$$

The reference point to compute the PRC is chosen to be when *V* crosses $V_{\mathrm{th}}$ in the positive direction. Note again that this method of computing the PRC is different from computing the iPRC of a spiking neuron which yields a strictly Type 1 PRC. The PRC we obtain is very similar, but there is a region of the PRC that is positive near small stimulus phases due to the longer active duration of the neuron.

#### 2.2.1 Selection of PRCs

In order for our analytical estimates to match the results of numerical simulations of the model, we took advantage of the computability of a PRC for the M–L neuron. In each iteration, we numerically computed the response of a neuron to a synaptic input of a specific strength at a specific phase. Although this method yields accurate results, it is computationally slow and it is almost impossible to implement on biological neurons. For this purpose, we created a meshed PRC measured at discrete phase points and for a discrete set of predetermined synaptic strengths. We used mesh sizes of 0.1 for the phase and 0.0125 for the synaptic strength to obtain a total of 77 points of numerically computed phase response values. The responses to the phases and strengths not on the mesh points were calculated by linear interpolation.

### 2.3 Model for Synaptic Plasticity

The short-term synaptic plasticity in spiking cells can be described by a phenomenological model [16]. We modify this model for neurons that have broader action potentials or those for which the burst envelope instead of individual spikes are modeled. To account for the longer durations that the neurons spend above the threshold we assume that there are two variables which determine the strength of the synapses when a neuron fires; the depression variable (*r*) and a facilitation variable (*u*). The depression variable *r* represents the amount of available synaptic resources, while the variable *u* represents the amount of utilized synaptic resources. They change according to the activity of the presynaptic cell and together determine the synaptic strength. These variables obey the following dynamics:

(2.3) $$\begin{array}{rl} \frac{dr}{dt} & =\{\begin{array}{cc} \frac{-r}{\tau_{1}}, & V\geq V_{\mathrm{th}}\\ \frac{1-r}{\tau_{2}}, & V<V_{\mathrm{th}} \end{array}\\ \frac{du}{dt} & =\{\begin{array}{cc} \frac{1-u}{\tau_{3}}, & V\geq V_{\mathrm{th}}\\ \frac{U-u}{\tau_{4}}, & V<V_{\mathrm{th}} \end{array} \end{array}$$

When the membrane voltage of the presynaptic cell is above the synaptic threshold $V_{\mathrm{th}}$, the depression variable *r* approaches 0 with time constant $\tau_{1}$, representing the depletion of available synaptic resources. During this time interval, the facilitation variable *u* approaches 1 with time constant $\tau_{3}$ representing the increase in utilized resources. When the membrane voltage is below the synaptic threshold, these variables recover to their steady-state values of 1 and *U*, with time constants $\tau_{2}$ and $\tau_{4}$, respectively. The strength of the synapses is determined by scaling the maximal synaptic conductance by the product of the values of these variables when the presynaptic cell crosses $V_{\mathrm{th}}$. If the presynaptic cell fires a sequence of spikes, then the term *n*th cycle refers to the time duration between the *n*th and n+1st crossings of $V_{\mathrm{th}}$. Hence, the synaptic conductance at the start of the *n*th cycle is given by $g_{\mathrm{pre}\to \mathrm{post}}={\bar{g}}_{\mathrm{pre}\to \mathrm{post}}r_{n}u_{n}$, where $r_{n}$ and $u_{n}$ are the values of *r* and *u* when the presynaptic membrane potential passes synaptic threshold in the *n*th cycle (*n* is defined below).

#### 2.3.1 Steady State Synaptic Plasticity Profiles

If the presynaptic cell fires with a fixed frequency, then it reaches an oscillatory steady state. The values *r* and *u* then also reach steady states and each oscillates between a minimum and a maximum value. At steady state, when crossing the synaptic threshold, $r_{n}$ is at a maximum, $r_{\max}$, while $u_{n}$ attains its minimum, $u_{\min}$. These values can be calculated from (2.3) as

(2.4) $$\begin{array}{rl} r_{\max} & =\frac{1-e^{-t_{b}/\tau_{2}}}{1-e^{-t_{a}/\tau_{1}}e^{-t_{b}/\tau_{2}}}\\ u_{\min} & =\frac{U+e^{-t_{b}/\tau_{4}}-e^{-t_{b}/\tau_{4}}(U+e^{-t_{a}/\tau_{3}})}{1-e^{-t_{a}/\tau_{3}}e^{-t_{b}/\tau_{4}}} \end{array}$$

where $t_{a}$ and $t_{b}$ are the durations that the cell spends above and below $V_{\mathrm{th}}$, respectively.

It is often possible to measure the strength of the synaptic output when the presynaptic neuron is driven in a range of frequencies. The values $r_{\max}$ and $u_{\min}$ depend on the presynaptic frequency and an appropriate choice of time constants allows for our model to fit a variety of frequency-dependent synaptic outputs. In particular, we are interested in synapses whose strength is maximal at a unique “preferred” frequency as we have observed in experimental measurements [19]. In our results presented below, we will use period instead of frequency for ease of analysis. By choosing appropriate parameters, therefore, we can match the period at which the peak of the product $r_{\max}u_{\min}$ is maximized with the experimentally measured preferred period of the synapse. We define the function

(2.5) $$g(P)=\bar{g}r_{\max}(P)u_{\min}(P)$$

as the synaptic strength at the time of firing of a presynaptic neuron with constant period $P=t_{a}+t_{b}$. We will assume that the changes in period of the bursting neurons affect only the inter-burst duration (i.e., $t_{a}$ is fixed). We will henceforth refer to this relationship (2.5) as the steady-state synaptic plasticity profile.

**Fig. 2:**
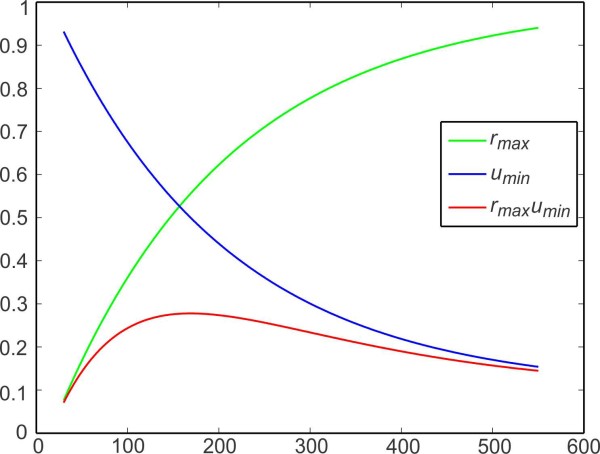
Steady-state values of plasticity variables. The maximum value $r_{\max}$ that the depression variable *r* and the minimum value $u_{\min}$ that the facilitation variable *u* reach at the steady state at the onset of presynaptic activity plotted against the presynaptic period. The plasticity profile of the synapse is given by their product

Figure 2 shows plots of the steady-state values of $r_{\max},u_{\min}$ and the full synaptic plasticity profile ($r_{\max}u_{\min}$) of a synapse as a function of the firing period, for a given set of parameters. Here $t_{a}=15$. The peak of the synaptic plasticity profile in this case occurs at P=170. For ease of analysis in Sect. 3.3 and thereafter, we use a Gaussian function approximation for the steady-state synaptic plasticity profile g(P):

(2.6) $$g(P)=0.75e^{-{\left(P-P_{\mathrm{pref}}\right)}^{2}/(2\sigma^{2})}+0.75$$

where $P_{\mathrm{pref}}$ is the peak of the profile corresponding to the preferred period of the synapse and *σ* determines the spread. 

## 3 Results

We derive Poincaré maps that relate the firing times of a network of two neurons coupled with reciprocal inhibition. We assume a predetermined one-to-one firing order between the neurons. The fixed points of these maps correspond to one-to-one firings of the neurons at the steady state. It is possible to derive similar maps assuming orders of firing that are not one-to-one, but these derivations are beyond the scope of the current study. We first assume a fixed synaptic strength between the neurons in Sect. 3.1. When the synapses have a fixed strength, only the phase response information of the neurons is used to determine the network activity, as has been shown previously [8]. In Sect. 3.2 we derive maps that describe the network activity when the synapses between the neurons are plastic. We compare two cases. In one case, we assume that the synapses obey the plasticity dynamics given in Eq. (2.3). In the second case, we consider synapses that obey the corresponding steady-state values given in Eq. (2.4). The latter case results in a lower-dimensional map. In Sect. 3.3, assuming both synapses obey a steady-state plasticity profile (2.6), we examine how changes in these profiles determine the network period and relative phase relations. We find conditions for a network to be able to keep a fixed firing period but vary the relative firing phase between its neurons, and vice versa.

### 3.1 Map for Phase with Static Synaptic Strength

We start with a network of two oscillatory neurons reciprocally inhibiting each other with constant synaptic strength. We will derive a 1D map that measures the phase difference between the onset of firing of the two cells. A fixed point of the map corresponds to a 1:1 phase-locked solution. We then derive the criteria for existence and stability of fixed points. Finally, we test the map in a network of two M–L model neurons.

**Fig. 3:**
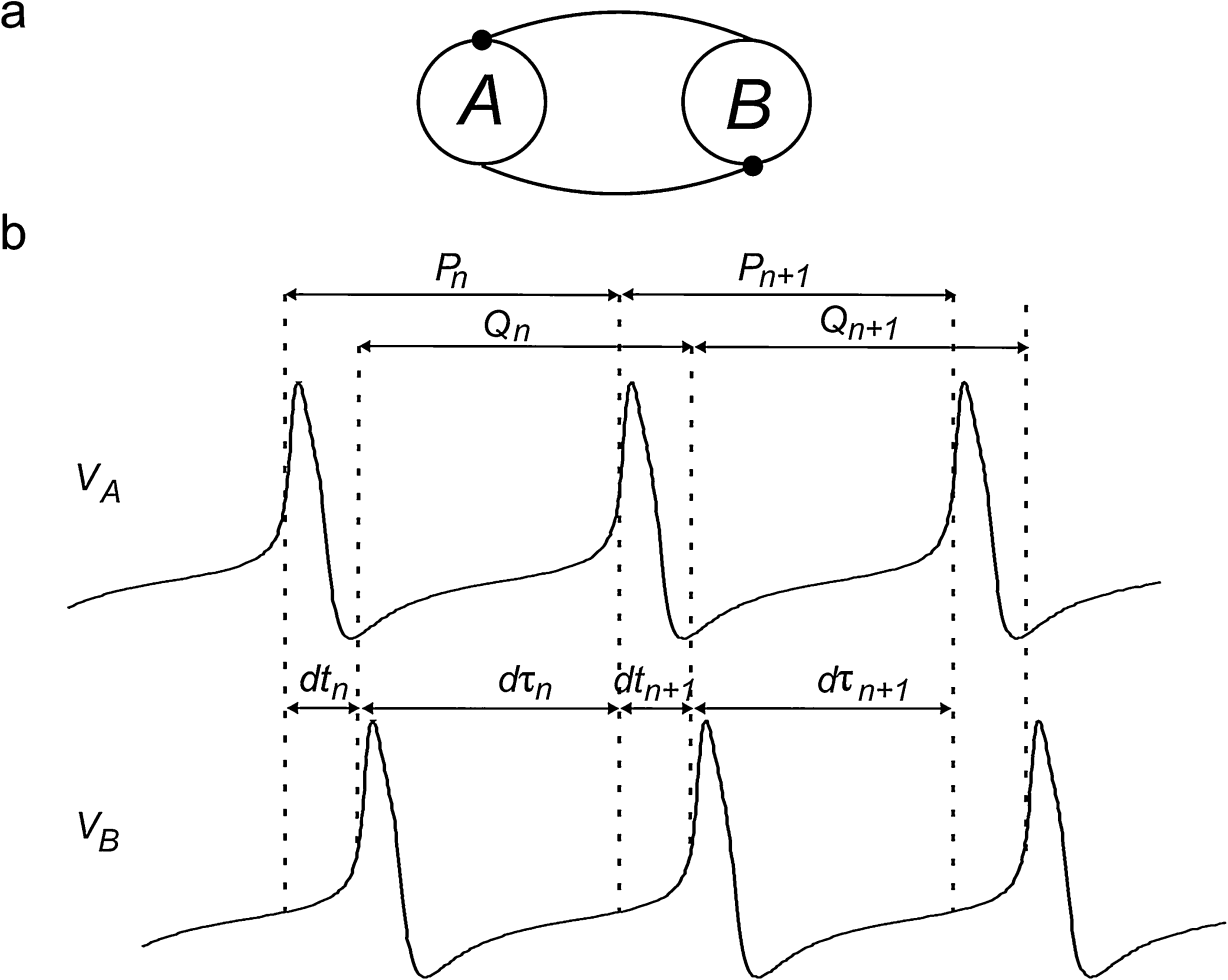
Schematic diagram of the coupled network and the map variables. **a** Schematic of the network connectivity diagram. **b** The cycle length $P_{n}$ of cell A in cycle *n* (measured for the M–L simulations when voltage crosses $V_{\mathrm{th}}$) can be divided into the delay between cell A activity to cell B activity ($dt_{n}$) and the opposite ($d\tau_{n}$). The cycle period $Q_{n}$ of cell B in cycle *n* is $d\tau_{n}+dt_{n+1}$

Consider a network of two oscillatory cells, A and B, coupled with reciprocal inhibition (Fig. 3a). Assume that the synaptic strengths between the cells are constant in each spike, i.e., $g_{A\to B}=g_{B\to A}=\bar{g}$. The intrinsic period of cell A and cell B are denoted by $P_{0}$ and $Q_{0}$, respectively. When the neurons are synaptically coupled, the time between subsequent firing of the same neuron may change. This time is called the cycle length, denoted by $P_{n}$ and $Q_{n}$ in cycle *n*, respectively for A and B. 

We derive a Poincaré map for the relative firing times of the neurons when they are synaptically connected. We choose the Poincaré section to be at $V_{A}=V_{\mathrm{th}}$. The amount of time that passes after cell A fires until cell B fires is denoted by $dt_{n}$, while the amount of time after cell B until cell A fires is denoted by $d\tau_{n}$ (Fig. 3b). The (activity) phase of neuron A (or B) is defined as the firing time $dt_{n}$ (or $d\tau_{n}$) normalized by the cycle length. Therefore, the phases of A and B are, respectively, given by

(3.1a) $${\tilde{\phi }}_{n}=dt_{n}/P_{n}$$

(3.1b) $${\tilde{\theta }}_{n}=d\tau_{n}/Q_{n}$$

In the derivations of the maps, we will make use of the PRCs of A and B which are defined in terms of $P_{0}$ and $Q_{0}$, the intrinsic periods of A and B. To simplify these derivations we introduce the notation of the “intrinsic phase” of neurons A and B which are defined, respectively, as

(3.2a) $$\phi_{n}=dt_{n}/P_{0}$$

(3.2b) $$\theta_{n}=d\tau_{n}/Q_{0}$$

We denote the PRC of cell A and cell B as $Z_{A}(\cdot )$ and $Z_{B}(\cdot )$, respectively, for synaptic inputs with a fixed strength. Rewriting the PRC relationship (2.2) for the cycle lengths, we can obtain the cycle lengths of each cell in cycle *n* as

(3.3a) $$P_{n}=P_{0}\left(1-Z_{A}(\phi_{n})\right)$$

(3.3b) $$Q_{n}=Q_{0}\left(1-Z_{B}(\theta_{n})\right)$$

The following equations relate the firing times of the two cells:

(3.4a) $$dt_{n}+d\tau_{n}=P_{n}$$

(3.4b) $$d\tau_{n}+dt_{n+1}=Q_{n}$$

From (3.3a) and (3.4a), $\theta_{n}$ can be written in terms of $\phi_{n}$:

(3.5) $$\begin{array}{rl} \theta_{n} & =\frac{d\tau_{n}}{Q_{0}}=\frac{1}{Q_{0}}(P_{n}-dt_{n})=\frac{1}{Q_{0}}\left[P_{0}\left(1-Z_{A}(\phi_{n})\right)-P_{0}\phi_{n}\right]\\ & =\frac{P_{0}}{Q_{0}}\left(1-Z_{A}(\phi_{n})-\phi_{n}\right) \end{array}$$

Similarly, $\phi_{n+1}$ can be expressed in terms of $\theta_{n}$:

(3.6) $$\begin{array}{rl} \phi_{n+1} & =\frac{dt_{n+1}}{P_{0}}=\frac{1}{P_{0}}(Q_{n}-d\tau_{n})=\frac{1}{P_{0}}\left[Q_{0}\left(1-Z_{B}(\theta_{n})\right)-\theta_{n}Q_{0}\right]\\ & =\frac{Q_{0}}{P_{0}}\left(1-Z_{B}(\theta_{n})-\theta_{n}\right) \end{array}$$

using (3.3b) and (3.4b).

Thus, plugging Eq. (3.5) into Eq. (3.6) defines the following 1D map for the intrinsic phase of cell A (3.2a) when the 1:1 firing order between the cells is maintained:

(3.7) $$\begin{array}{rl} \phi_{n+1} & =\Pi (\phi_{n})\\ & =\frac{Q_{0}}{P_{0}}\left[1-Z_{B}\left(\frac{P_{0}}{Q_{0}}\left(1-Z_{A}(\phi_{n})-\phi_{n}\right)\right)\right]-1+Z_{A}(\phi_{n})+\phi_{n} \end{array}$$

The condition for a 1:1 phase-locking solution is $\phi_{n}=\phi_{n+1}=\phi^{*}$. Plugging this into the map gives the condition for a fixed point as

(3.8) $$P_{0}\left(1-Z_{A}\left(\phi^{*}\right)\right)=Q_{0}\left(1-Z_{B}\left(\theta^{*}\right)\right)$$

where $\theta^{*}=\frac{P_{0}}{Q_{0}}(1-Z_{A}(\phi^{*})-\phi^{*})$. The fixed point is stable if $|\Pi^{\prime }(\phi^{*})|<1$, hence the stability condition is

(3.9) $$\left|\left(Z_{A}^{\prime }\left(\phi^{*}\right)+1\right)\left(Z_{B}^{\prime }\left(\theta^{*}\right)+1\right)\right|<1$$

This result was previously found in [8]. If the neurons are identical, $P_{0}=Q_{0}$ and $Z_{A}(\cdot )=Z_{B}(\cdot )=Z(\cdot )$. Then the map (3.7) reduces to

(3.10) $$\begin{array}{rl} \phi_{n+1} & =\Pi (\phi_{n})\\ & =-Z\left(1-Z(\phi_{n})-\phi_{n}\right)+Z(\phi_{n})+\phi_{n} \end{array}$$

The fixed point equation (3.8) becomes

(3.11) $$Z\left(\phi^{*}\right)=Z\left(1-Z\left(\phi^{*}\right)-\phi^{*}\right)$$

and the stability condition (3.9) becomes

$$\left|\left(Z^{\prime }\left(\phi^{*}\right)+1\right)\left(Z^{\prime }\left(1-Z\left(\phi^{*}\right)-\phi^{*}\right)+1\right)\right|<1$$

In this symmetric case, the phase locking of the network does not depend on the intrinsic periods $P_{0}$ of the network neurons. The phase of cell A (3.1a) in cycle *n* can be obtained from the relation

$${\tilde{\phi }}_{n}=\frac{dt_{n}}{P_{n}}=\frac{\phi_{n}P_{0}}{P_{n}}$$

which can be simplified using Eq. (3.3a) to

(3.12) $${\tilde{\phi }}_{n}=\frac{\phi_{n}}{1-Z(\phi_{n})}\equiv f(\phi_{n})$$

Given the map (3.10) for $\phi_{n}$, in order to derive a map for ${\tilde{\phi }}_{n+1}$, we need the function given in (3.12) to be invertible. The function *f* is monotone increasing in [0,1] if and only if $f^{\prime }(\phi )\geq 0$ on this interval where

$$f^{\prime }(\phi )=\frac{1-Z(\phi )+\phi Z^{\prime }(\phi )}{{\left(1-Z(\phi )\right)}^{2}}$$

**Fig. 4:**
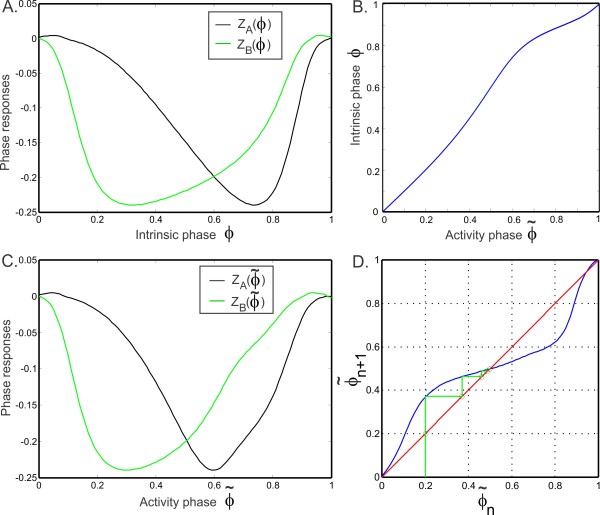
Phase locking for static synapses. **a** The left and right hand sides of the fixed point equation (3.11) for two identical neurons. The left hand side (*black*) is the response of neuron A and the right hand side is the response of neuron B at steady state. The intersection gives the fixed point. Note that *the black curve* is the PRC of both neurons. **b** The relation $f^{-1}$ between the intrinsic phase *ϕ* (3.2a) and the activity phase $\tilde{\phi }$ (3.1a). **c** The same graph as panel **a** plotted as functions of the activity phase $\tilde{\phi }$ using the transformation from *ϕ* to $\tilde{\phi }$ shown in panel **b**. **d** Convergence of the iterates starting with the initial condition ${\tilde{\phi }}_{0}=0.2$ is shown in a cobweb diagram. The iterates (in *green*) converge to the fixed point at the intersection of the graph of ${\tilde{\phi }}_{n+1}=\Pi ({\tilde{\phi }}_{n})$ with the line ${\tilde{\phi }}_{n}={\tilde{\phi }}_{n+1}$

The denominator is always positive. The numerator is positive if $Z^{\prime }(\phi )\geq 0$. For a standard Type 1 PRC (with a single local extremum), this will occur if *ϕ* is large enough (i.e., larger than the minimum point of the PRC; see Fig. 1b). For our choice of parameters this occurs when ϕ>0.75 (Fig. 1b) where the PRC is increasing. On the remaining interval, the expression 1−Z(ϕ) is ≥1. So if $Z^{\prime }(\phi )\geq -1/\phi \geq -4/3$ on [0,0.75], then $f^{\prime }(\phi )$ would also be positive and *f* could then be inverted on [0,1] (Fig. 4b). However, it is not possible to analytically make this estimate since we have no closed form expression for Z(ϕ). We confirmed numerically though that $Z^{\prime }(\phi )\geq -4/3$ in this interval, hence $f^{\prime }(\phi )$ is positive on [0,1]. Therefore, the function *f* can be inverted on [0,1]. The numerically obtained inverse function $f^{-1}$ is shown in Fig. 4b. Hence, the phase of cell A (3.1a) in cycle n+1 can be obtained from its value in cycle *n* from

(3.13) $${\tilde{\phi }}_{n+1}=f\left(\Pi \left(f^{-1}({\tilde{\phi }}_{n})\right)\right)\equiv \tilde{\Pi }({\tilde{\phi }}_{n})$$

In general, the function *f* (3.12) and the map $\tilde{\Pi }$ (3.13) can be defined for networks consisting of either identical or non-identical neurons. Here we have considered only the networks of identical neurons in this section. The generalization to networks of non-identical neurons is considered below in Sect. 3.3.3.

We can now assess the existence and stability of fixed points of the maps (3.10) and (3.13). We numerically solved the map (3.10) using MATLAB to predict the activity of two identical M–L neurons coupled with reciprocal inhibition. We also numerically solved the differential equations governing the activity of the neurons using XPPAUT [20]. We let the maximal synaptic conductance $\bar{g}$ equal 0.1 and use the PRCs of the neurons obtained for this value of synaptic strength. We first find the fixed points of the map by solving the fixed point equation (3.11). The two sides of Eq. (3.11) are plotted in Fig. 4a. They intersect only at one point $\phi^{*}=0.598$, which corresponds to the intrinsic phase of cell A (3.2a) at the steady state. The firing period of cell A can be obtained from Eq. (3.3a) evaluated at this intrinsic phase. This value is also equal to the period of B and will be referred to as the period of the coupled network ($P_{\mathrm{st}}$). The activity phase ${\tilde{\phi }}^{*}$ of cell A (3.1a) at the steady state is 0.5 and is obtained by using (3.12), corresponding to the anti-phase solution, which agrees with the simulations (not shown). In Fig. 4c, the right and left hand sides of the fixed point equation (3.11) are plotted as functions of the activity phase using (3.12). They intersect at ${\tilde{\phi }}^{*}=0.5$. In Fig. 4d, we show the cobweb diagram for the map (3.13), starting with the initial condition ${\tilde{\phi }}_{0}=0.2$ leading to convergence to the stable steady state of ${\tilde{\phi }}^{*}=0.5$. For this case, the system locks in the anti-phase state because the two neurons and the two synaptic strengths are identical.

### 3.2 Maps Using Dynamic Synapses or Steady-State Synaptic Plasticity Profiles in One Synapse

In this section we derive maps to predict the network activity in the presence of synaptic plasticity. We now let the synaptic strength from cell A to cell B be constant and the strength from cell B to cell A exhibit plasticity.

**Fig. 5:**
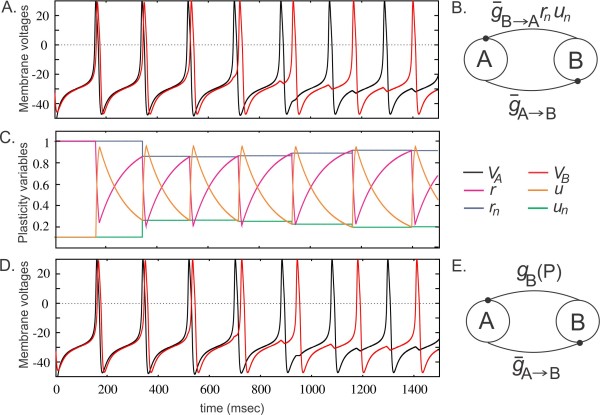
Two-cell network with synaptic plasticity in one synapse. **a** Voltage traces obtained from simulations of the M–L neurons when the A to B synapse is of fixed strength and B to A synapse changes according to the plasticity model (2.3). **c** The evolution of the plasticity variables *r*, *u*, $r_{n}$, and $u_{n}$ according to the activity of neuron B. **d** Voltage traces obtained from simulations of the M–L neurons when the A to B synapse is of fixed strength and B to A synapse changes according to the steady-state plasticity profiles given by (2.4). **b** &**e** Network connectivity diagram corresponding to the simulations shown in **a** &**d**. The parameter values for the plasticity variables are $\tau_{1}=2$, $\tau_{2}=190$, $\tau_{3}=2$, $\tau_{4}=190$

The correct method for deriving the map is to assume that the strength of the synapse from B to A changes according to plasticity dynamics given in (2.3). However, often in experiments it is easy to measure the steady-state response of a synapse at different input frequencies without knowing what the underlying dynamics are that give rise to this steady-state value. That is, it is possible to measure the steady-state synaptic plasticity profile g(P) obtained from Eq. (2.5). We therefore consider two different approaches in the derivation of the map. In the first derivation we assume that the strength of the B to A synapse is determined by the plasticity dynamics given in (2.3), whereas, in the second approach, we assume that the strength of this synapse obeys the steady-state synaptic plasticity profile $g_{B}(P)$ given by (2.5) (Figs. 5b and 5e). The first approach allows the transients due to different initial conditions to potentially play a role in the convergence of the map to a fixed point. We show, however, that both approaches produce the same result. 

When plasticity is included in the B to A synapse, the synaptic strength is no longer constant. Hence we cannot use a unique PRC for neuron A. Instead, we define a PRC as a function of two variables, where the phase at which the synapse is received and the strength of the synapse determine the response of the neuron. We denote this by $Z_{A}(\phi ,g)$. The PRC of neuron B is obtained for a constant synaptic strength ${\bar{g}}_{A\to B}$ and is denoted by $Z_{B}(\theta )$.

We will now determine the phase of neuron A and the network period for the two models where the B to A synapse either 

i. changes according to the dynamics of the plasticity variables *r* and *u* and is given by ${\bar{g}}_{B\to A}r_{n}u_{n}$, or,

ii. obeys the steady-state synaptic plasticity profile $g_{B}(P)={\bar{g}}_{B\to A}r_{\max}(P)u_{\min}(P)$.

We start with the derivation of the map using the dynamics of plasticity variables (case i). The voltage traces of the neurons A and B and the evolution of the plasticity variables of neuron B obtained from simulations are shown in Figs. 5a and 5c, respectively. In this case, the response of neuron A in cycle *n* depends on the values of the plasticity variables in this cycle. Assume that we know the values $\phi_{n}$, $r_{n}$ and $u_{n}$. Then we can compute the period of neuron A in cycle *n* using the expression

(3.14) $$P_{n}=P_{0}\left(1-Z_{A}(\phi_{n},{\bar{g}}_{B\to A}r_{n}u_{n})\right)$$

We can next modify Eq. (3.5) by rewriting $P_{n}$ as given in (3.14) to obtain the intrinsic phase of neuron B (3.2b) in cycle *n* as

$$\theta_{n}=\frac{P_{0}}{Q_{0}}\left(1-Z_{A}(\phi_{n},{\bar{g}}_{B\to A}r_{n}u_{n})-\phi_{n}\right)$$

The equation giving the cycle length of neuron B becomes

(3.15) $$Q_{n}=Q_{0}\left(1-Z_{B}\left(\frac{P_{0}}{Q_{0}}\left(1-Z_{A}(\phi_{n},{\bar{g}}_{B\to A}r_{n}u_{n})-\phi_{n}\right)\right)\right)$$

in cycle *n*. Using Eq. (3.7) together with the above equations gives a 3D map for the evolution of the intrinsic phase of cell A (3.2a) and the synaptic plasticity variables from cell B to cell A

(3.16) $$\begin{array}{rl} \phi_{n+1}= & \frac{Q_{0}}{P_{0}}\left[1-Z_{B}\left(\frac{P_{0}}{Q_{0}}\left(1-Z_{A}(\phi_{n},{\bar{g}}_{B\to A}r_{n}u_{n})-\phi_{n}\right)\right)\right]\\ & -1+Z_{A}(\phi_{n},{\bar{g}}_{B\to A}r_{n}u_{n})+\phi_{n}\\ r_{n+1}= & 1-\left(1-r_{n}e^{-t_{a}/\tau_{1}}\right)\\ & \times \exp[-(Q_{0}\left(1-Z_{B}\left(\frac{P_{0}}{Q_{0}}\left(1-Z_{A}(\phi_{n},{\bar{g}}_{B\to A}r_{n}u_{n})-\phi_{n}\right)\right)\right)\\ & -t_{a})/\tau_{2}]\\ u_{n+1}= & U-\left(U-1+(1-u_{n})e^{-t_{a}/\tau_{3}}\right)\\ & \times \exp[-(Q_{0}\left(1-Z_{B}\left(\frac{P_{0}}{Q_{0}}\left(1-Z_{A}(\phi_{n},{\bar{g}}_{B\to A}r_{n}u_{n})-\phi_{n}\right)\right)\right)\\ & -t_{a})/\tau_{4}] \end{array}$$

The first equation is the same as (3.7) except that now $Z_{A}$ is a function of two arguments. The second and third equations are computed using (2.3) over one cycle. The complicated expressions in the exponential of both equations are the time $Q_{n}-t_{a}$ recast in terms of $\phi_{n},r_{n},u_{n}$ where $Q_{n}$ is given in Eq. (3.15).

We next derive the map for case ii where the synapse from neuron A to neuron B has a constant strength at each cycle, while the synaptic strength from neuron B to A changes according to the steady-state plasticity function $g_{B}(x)$. The voltage traces of the neurons A and B obtained from simulations are shown in Fig. 5d. In this case, instead of the depression and facilitation variables, we can use the cycle length of one of the neurons to derive the activity map. We assume that we know the values $\phi_{n}$ and $P_{n}$. Then the intrinsic phase of neuron B (3.2b) in cycle *n* can be found by using (3.4a) as

(3.17) $$\theta_{n}=(P_{n}-\phi_{n}P_{0})/Q_{0}$$

Plugging this into (3.3b) immediately yields the expression for the cycle length of neuron B in cycle *n* as

(3.18) $$Q_{n}=Q_{0}\left[1-Z_{B}\left((P_{n}-\phi_{n}P_{0})/Q_{0}\right)\right]$$

We can now obtain the intrinsic phase of neuron A (3.2a) in cycle n+1 using Eq. (3.4b) as

(3.19) $$\phi_{n+1}=(Q_{n}-d\tau_{n})/P_{0}=(Q_{n}-\theta_{n}Q_{0})/P_{0}$$

We use this phase to obtain the cycle length of neuron A in cycle n+1 as

(3.20) $$P_{n+1}=P_{0}\left[1-Z_{A}\left(\phi_{n+1},g_{B}(Q_{n})\right)\right]$$

Similar to Eq. (3.14), the period of neuron A is determined by $Z_{A}$ which is a function of two variables. However, in this case the synaptic strength received by neuron A in cycle n+1 depends directly on the cycle length of neuron B in cycle *n*.

The map for the activity of the network can be obtained by plugging (3.17) and (3.18) into (3.19) and (3.20) as

(3.21) $$\begin{array}{rl} \phi_{n+1}= & \frac{Q_{0}}{P_{0}}\left[1-Z_{B}\left(\frac{P_{n}-\phi_{n}P_{0}}{Q_{0}}\right)\right]-\frac{P_{n}}{P_{0}}+\phi_{n}\\ P_{n+1}= & P_{0}[1-Z_{A}(\frac{Q_{0}}{P_{0}}\left[1-Z_{B}\left(\frac{P_{n}-\phi_{n}P_{0}}{Q_{0}}\right)\right]-\frac{P_{n}}{P_{0}}+\phi_{n},\\ & g_{B}\left(Q_{0}\left[1-Z_{B}\left(\frac{P_{n}-\phi_{n}P_{0}}{Q_{0}}\right)\right]\right))] \end{array}$$

Hence, the map (3.16) is reduced to a 2D map for the intrinsic phase and cycle length of neuron A. A fixed point $\left(\phi^{*},r^{*},u^{*}\right)$ of the 3D map (3.16) corresponds to a 1:1 solution. This 1:1 solution is also represented by a fixed point of the 2D map (3.21) which occurs at $\left(\phi^{*},P^{*}\right)$, where $P^{*}$ is the steady-state value obtained from (3.17) at $\left(\phi^{*},r^{*},u^{*}\right)$.

To assess numerically the existence and stability of the fixed points of both the 2D map (3.21) and the 3D map (3.16), consider two identical neurons coupled with asymmetric synapses. Let the synaptic strength from neuron A to B be fixed at ${\bar{g}}_{A\to B}=0.1$. We use parameters for the plasticity variables that yield the steady-state plasticity function $g_{B}(P)$ with a peak at the period 169.5, as shown in Fig. 2. Denote the steady-state network period and phase of neuron A from the 3D map (case i) as $P_{\mathrm{dyn}}$ and $\phi_{\mathrm{dyn}}$, respectively, and the corresponding values from the 2D map (case ii) as $P_{\mathrm{ss}}$ and $\phi_{\mathrm{ss}}$. Similarly, for static coupling, denote the steady-state network period as $P_{\mathrm{st}}$ and phase of neuron A as $\phi_{\mathrm{st}}$.

**Fig. 6:**
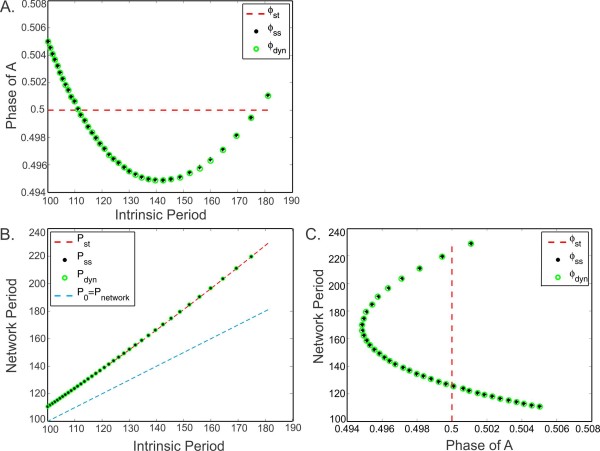
A comparison of the 1D (3.7), 2D (3.21) and 3D (3.16) maps. **a** The steady-state phase of the neuron A, $\phi_{\mathrm{st}}$ from map (3.7), $\phi_{\mathrm{dyn}}$ from map (3.16), $\phi_{\mathrm{ss}}$ from map (3.21), shown as a function of the intrinsic period of both neurons (changed simultaneously). **b** The network period as a function of intrinsic periods corresponding to the same maps. **c** The relation between the network period and the phase of A for the same maps. The phase of A reaches a minimum at the network period equal to the preferred period of neuron B. The results of the two maps with plasticity ((3.14) and (3.19)) overlap in all panels

Figure 6 shows the steady-state phase of neuron A and the network period obtained from the 1D map (3.7), the 3D map (3.16) and the 2D map (3.21), for a set of intrinsic periods $P_{0}$ (varied simultaneously in both cells). In Fig. 6a, the steady-state phase of neuron A is plotted as a function of $P_{0}$. The maps with plasticity (cases i and ii) yield the same steady-state phase of neuron A; this phase is not constant but is a function of the intrinsic period (green and black), in contrast to the static case where the network always has an anti-phase solution (dashed red line). This variation in phase depends on the values of the steady-state plasticity profile $g_{B}(P)$ (further explained below). Figure 6b compares the steady-state network period obtained from the three maps. The periods obtained from the maps with plasticity are again the same and they are slightly different from the periods obtained from the static map. The blue dashed line is the $P_{0}=P_{\mathrm{network}}$ line. The network period is always larger than the intrinsic period in all cases, due to the selection of the PRC (that the inhibitory input always delays the next firing time). Figure 6c relates the steady-state phase of neuron A with the network period. 

We now examine how the steady-state phase of neuron A changes with respect to changes in the intrinsic period. The phase of neuron A depends on the value of the synaptic strength received from neuron B at the steady state. This value is determined by $Q^{*}$, the steady-state firing period of neuron B, which equals the steady-state network period $P^{*}$. When this value equals ${\bar{g}}_{A\to B}=0.1$, then anti-phase solutions occur. This happens for two sets of coupled neurons, where the red dashed line intersects green and black curves (Figs. 6a and 6c). Between these two points, the synaptic strength received by neuron A, given by $g_{B}(Q^{*})$, is larger than ${\bar{g}}_{A\to B}$. Since the cells are identical, the neurons must give equal amount of response (so that their steady-state firing periods will be equal) for a steady-state solution to occur. When both synaptic strengths are equal, both neurons have steady-state phase at 0.5. However, if $g_{B}(Q^{*})>{\bar{g}}_{A\to B}$, then neuron A receives stronger synaptic input than neuron B. This difference can be balanced if neuron A receives this synaptic input at a phase that yields less response. As the PRCs of the neurons are decreasing around the phase 0.5, neuron A needs to phase lock at a phase smaller than 0.5. This explains why phase of neuron A decreases between these intersection points. A similar argument holds when $g_{B}(Q^{*})<{\bar{g}}_{A\to B}$.

The phase of neuron A reaches a minimum when the synaptic strength reaches a maximum. As can be seen in Fig. 2, the synaptic plasticity profile has its peak at 169.5. Therefore, the minimum phase of neuron A is observed at the network period 169.5 (Fig. 6c). The network period of 169.5 is obtained when two cells with intrinsic periods 141.8 are coupled (Fig. 6b).

### 3.3 Maps Using Steady-State Synaptic Plasticity Profiles in Both Directions

Let both reciprocal synapses have short-term plasticity. The map involving the synaptic plasticity variables (2.3) that generalizes (3.16) would now be 5D. But given the results from the previous section showing that the simplified map using the steady-state synaptic plasticity profiles provides the same stable output, we derive only the 2D map associated with the latter. We again start with the intrinsic phase $\phi_{n}$ (3.2a) and cycle length $P_{n}$ of neuron A in cycle *n*. Equation (3.17) can still be used to obtain the intrinsic phase of neuron B (3.2b), $\theta_{n}$, in cycle *n*. However, the cycle length of neuron B is now given by the equation

(3.22) $$Q_{n}=Q_{0}\left[1-Z_{B}\left(\theta_{n},g_{A}(P_{n})\right)\right]$$

in cycle *n*, since the synapse from neuron A to B also has plasticity and depends on $P_{n}$. The cycle length *P* and intrinsic phase *ϕ* of neuron A in cycle n+1 is given by

(3.23) $$\begin{array}{rl} \phi_{n+1}= & \Pi_{1}(\phi_{n},P_{n})=\frac{1}{P_{0}}(Q_{n}-P_{n}+P_{0}\phi_{n})\\ = & \frac{Q_{0}}{P_{0}}\left[1-Z_{B}\left(\frac{1}{Q_{0}}(P_{n}-P_{0}\phi_{n}),g_{A}(P_{n})\right)\right]-\frac{P_{n}}{P_{0}}+\phi_{n}\\ P_{n+1}= & \Pi_{2}(\phi_{n},P_{n})=P_{0}\left[1-Z_{A}\left(\phi_{n+1},g_{B}(Q_{n})\right)\right]\\ = & P_{0}[1-Z_{A}(\frac{Q_{0}}{P_{0}}\left[1-Z_{B}\left(\frac{1}{Q_{0}}(P_{n}-P_{0}\phi_{n}),g_{A}(P_{n})\right)\right]-\frac{P_{n}}{P_{0}}+\phi_{n},\\ & g_{B}\left(Q_{0}\left[1-Z_{B}\left(\frac{1}{Q_{0}}(P_{n}-P_{0}\phi_{n}),g_{A}(P_{n})\right)\right]\right))] \end{array}$$

Equation (3.23) determines the values of *P* and *ϕ* when both synapses have plasticity. In the case where the two cells are identical, $Z_{A}(\cdot )=Z_{B}(\cdot )=Z$, this map simplifies to

(3.24) $$\begin{array}{rl} \phi_{n+1}= & \Pi_{1}(\phi_{n},P_{n})=\frac{1}{P_{0}}(Q_{n}-P_{n}+P_{0}\phi_{n})\\ = & 1-Z\left(\frac{1}{P_{0}}(P_{n}-P_{0}\phi_{n}),g_{A}(P_{n})\right)-\frac{P_{n}}{P_{0}}+\phi_{n}\\ P_{n+1}= & \Pi_{2}(\phi_{n},P_{n})=P_{0}\left[1-Z\left(\phi_{n+1},g_{B}(Q_{n})\right)\right]\\ = & P_{0}[1-Z(1-Z\left(\frac{1}{P_{0}}(P_{n}-P_{0}\phi_{n}),g_{A}(P_{n})\right)-\frac{P_{n}}{P_{0}}+\phi_{n},\\ & g_{B}\left(P_{0}\left[1-Z\left(\frac{P_{n}}{P_{0}}-\phi_{n},g_{A}(P_{n})\right)\right]\right))] \end{array}$$

We now explore whether these equations yield stable fixed points and, if so, how changes in the synaptic profiles affect the resulting phase- and period-locking of the network.

**Fig. 7:**
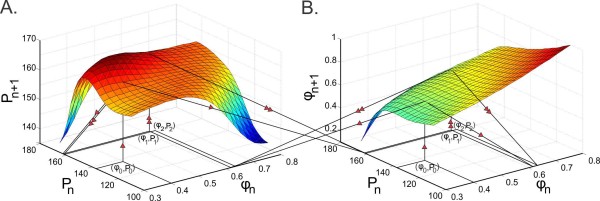
Cobwebbing diagram of the 2D map (3.24) for two identical cells ($P_{0}=Q_{0}$) and distinct synaptic plasticity profiles ($P_{A}=150$, $P_{B}=190$) shown in two coordinate systems. The period $P_{1}$ and the intrinsic phase $\phi_{1}$ of neuron A in cycle 1 is obtained by evaluating the initial condition $\left(\phi_{0},P_{0}\right)$ on the period surface $P_{n+1}=\Pi_{2}(\phi_{n},P_{n})$ (**a**) and the phase surface $\phi_{n+1}=\Pi_{1}(\phi_{n},P_{n})$ (**b**). The point $\left(\phi_{1},P_{1}\right)$ is then projected back to the *x*–*yaxis* in both coordinate systems and mapped to the point $\left(\phi_{2},P_{2}\right)$ with the same procedure. *Lines with one arrow* correspond to the first and *lines with two arrows* correspond to the second iteration

To be able to have explicit control of the preferred frequency of the synapses, instead of using Eq. (2.5) for g(P), we assume that the steady-state synaptic profiles obey Gaussian functions $g_{A}(\cdot )$ (for the A to B synapse) and $g_{B}(\cdot )$ (for the B to A synapse) (2.6) with peaks (preferred periods) $P_{A}$ and $P_{B}$, respectively. Equations (3.24) define two surfaces $\Pi_{1}(\phi_{n},P_{n})$ and $\Pi_{2}(\phi_{n},P_{n})$ which can be plotted in $R^{3}$. We plot two 3D coordinate systems to be able to visualize the evolution of the 2D map. We show three iterations of the map (3.24) in Fig. 7. The values $\left(\phi_{n},P_{n}\right)$ in cycle *n* are located on the *x*–*y* axes. These values are mapped through the surfaces $P_{n+1}=\Pi_{2}(\phi_{n},P_{n})$ (Fig. 7a) and $\phi_{n+1}=\Pi_{1}(\phi_{n},P_{n})$ (Fig. 7b) to the next iteration points $\left(\phi_{n+1},P_{n+1}\right)$ in cycle n+1. Start with the initial condition $\left(\phi_{0},P_{0}\right)$ which is shown in both coordinate systems. The image of $\left(\phi_{0},P_{0}\right)$ on the surface $\phi_{n+1}=\Pi_{1}(\phi_{n},P_{n})$ gives the next intrinsic phase value $\phi_{1}$, and the image of $\left(\phi_{0},P_{0}\right)$ on the surface $P_{n+1}=\Pi_{2}(\phi_{n},P_{n})$ gives the next cycle length $P_{1}$ (shown by the vertical lines with one arrow). These $\phi_{1}$ and $P_{1}$ values are located, respectively, on the *x* and *y* axes of both coordinate systems (shown by the inclined lines with one arrow). The point $\left(\phi_{1},P_{1}\right)$ is then located on the *x*–*y* axes in both coordinate systems and mapped to the point $\left(\phi_{2},P_{2}\right)$ by the same procedure (shown by the lines with two arrows). We are able to geometrically observe the iterations (only three shown) approach a fixed point; hence this is a generalization of cobwebbing for the 2D map. 

The fixed point equations of the map (3.24) in a 1:1 firing condition are

(3.25) $$\begin{array}{rl} P^{*}= & Q_{0}\left[1-Z_{B}\left(\frac{P^{*}-P_{0}\phi^{*}}{Q_{0}},g_{A}\left(P^{*}\right)\right)\right]\\ P^{*}= & P_{0}[1-Z_{A}(\frac{Q_{0}}{P_{0}}\left[1-Z_{B}\left(\frac{P^{*}-P_{0}\phi^{*}}{Q_{0}},g_{A}\left(P^{*}\right)\right)\right]-\frac{P^{*}}{P_{0}}+\phi^{*},\\ & g_{B}\left(Q_{0}\left[1-Z_{B}\left(\frac{P^{*}-P_{0}\phi^{*}}{Q_{0}},g_{A}\left(P^{*}\right)\right)\right]\right))] \end{array}$$

These simplify to

(3.26) $$\begin{array}{rl} P^{*} & =P_{0}\left[1-Z\left(\frac{P^{*}}{P_{0}}-\phi^{*},g_{A}\left(P^{*}\right)\right)\right]\\ P^{*} & =P_{0}\left[1-Z\left(\phi^{*},g_{B}\left(P^{*}\right)\right)\right] \end{array}$$

for identical cells.

**Fig. 8:**
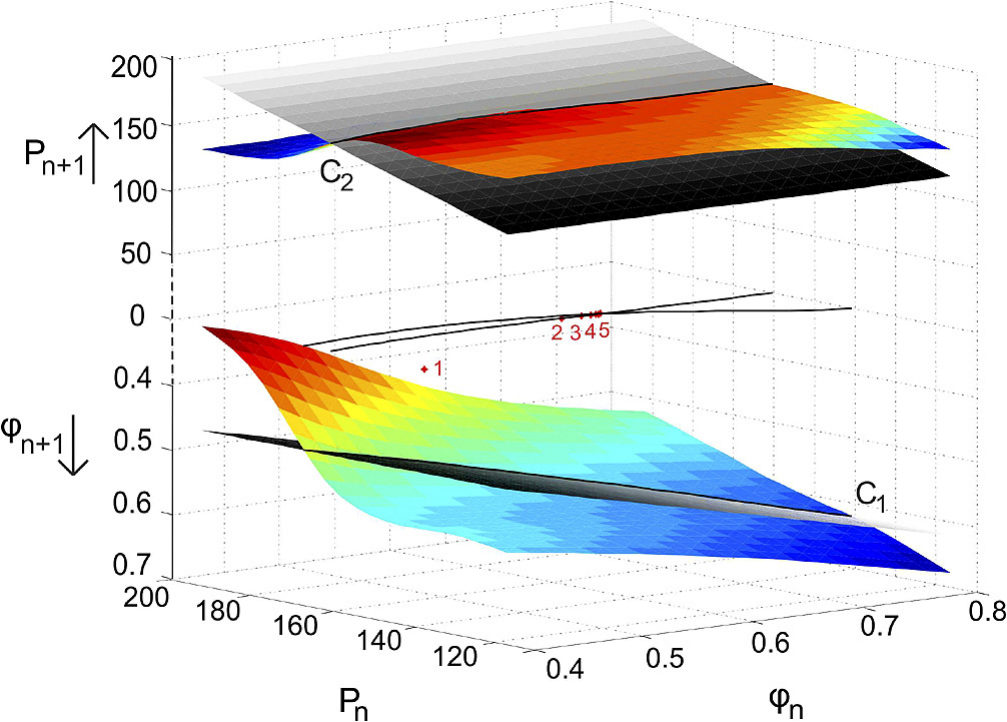
Fixed points of 2D (3.24) map when $P_{0}=Q_{0}$ obtained by solving (3.26). The surfaces for the evolution of period and intrinsic phase of the 2D map with synaptic preferred periods $P_{A}=150$, $P_{B}=190$ are drawn above and below the z=0 plane denoted by the axes $z_{1}=P_{n+1}$ and $z_{2}=\phi_{n+1}$, respectively. The equality $P_{n}=P_{n+1}$ is satisfied when the surface $z_{1}=\Pi_{2}(x,y)$ (*colored surface on top*) and the plane $z_{1}=y$ (*gray-scaled plane on top*) intersect. Similarly, the equality $\phi_{n}=\phi_{n+1}$ is satisfied when the surface $z_{2}=\Pi_{1}(x,y)$ (*colored surface on bottom*) intersects the plane $z_{2}=x$ (*gray-scaled plane on bottom*). These intersections yield *the two black curves above and below* the z=0 plane. The fixed point of the map lies on the intersection of the two fixed point curves. The projections of these curves on the z=0 plane are shown together with the iterates (*red dots*) approaching the fixed point at their intersection in the order enumerated in the *figure*

The fixed point of this 2D map occurs when $\phi_{n}=\phi_{n+1}$ and $P_{n}=P_{n+1}$. We can visualize how the fixed point is obtained. For this purpose, we plot the surfaces for the evolution of intrinsic phase and period (previously drawn on separate coordinate axes in Fig. 7) on the same coordinate axis, above and below the z=0 plane, and denote by the axes $z_{1}$ and $z_{2}$, respectively in Fig. 8. The equality $\phi_{n}=\phi_{n+1}$ is satisfied when the surface $z_{1}=\Pi_{1}(x,y)$ and the plane $z_{1}=x$ intersect. Denote this intersection curve as $C_{1}$. Similarly, the equality $P_{n}=P_{n+1}$ is satisfied when the surface $z_{2}=\Pi_{2}(x,y)$ intersects the plane $z_{2}=y$ (denoted as $C_{2}$). The curves $C_{1}$ and $C_{2}$ are shown in black above and below the z=0 plane. The fixed point of the map lies on both curves; hence it lies on the intersection of $C_{1}$ and $C_{2}$. The projections of $C_{1}$ and $C_{2}$ on the z=0 plane are shown in the figure together with the iterations (red dots) approaching the fixed point at their intersection. 

The stability of the fixed point can be examined using the Jacobian of the 2D map (3.24). If the eigenvalues of the Jacobian at the fixed point are located inside the unit circle, the fixed point is stable. For our choice of parameter values, the fixed point can be shown to be stable.

#### 3.3.1 Phase and Period Locking for Different Synaptic Plasticity Profiles

Having determined a method for calculating the steady-state network period and phase, we now determine how these quantities depend on various network parameters. For simplicity, in this section we consider identical neurons. We use the 2D map (3.24) to obtain the network phase and period when both synapses have plasticity. For comparison, we also obtain the same from the 1D map (3.10), when the synaptic strength is fixed.

**Fig. 9:**
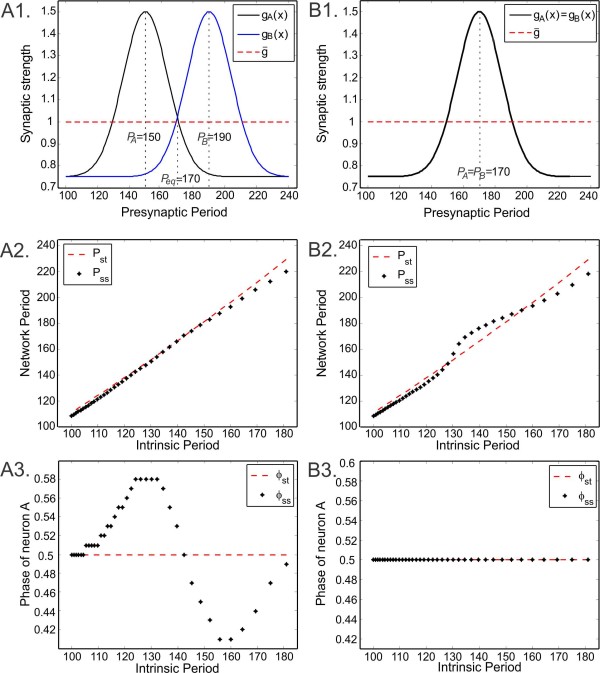
Period and phase locking when both synapses follow the synaptic plasticity profile. *Dashed line* in *all panels* shows the case with two static synapses. **a1** Synaptic plasticity profiles of the two synapses chosen to have different preferred periods at 150 and 190. **a2** Network period as a function of the intrinsic periods. **a3** Phase $\tilde{\phi }$ of neuron A with respect to B as a function of intrinsic period. **b1**–**b3** Same as **a1**–**a3** but with identical synaptic plasticity profiles (preferred period at 170)

We are interested in how differences in the plasticity profiles of the two synapses affect the network period and phase of neuron A (Figs. 9a1 and 9b1). The distinct plasticity profiles (Fig. 9a1) are produced by simply shifting one profile along the intrinsic period axis. In the non-identical case, the plasticity profiles are chosen to approach the same value at the tails (Fig. 9a1) and, therefore, for small (and large) intrinsic periods, $\phi_{\mathrm{ss}}=0.5$ due to identical synaptic strengths (Fig. 9a3). As the intrinsic period is increased, the difference between $g_{A}(P)$ and $g_{B}(P)$ first increases until $P=P_{A}$ and then decreases to zero when $P=P_{\mathrm{eq}}$ (Fig. 9a1). This causes $\phi_{\mathrm{ss}}$ to increase from 0.5 to 0.58 until $P_{\mathrm{ss}}=P_{A}$ and then decrease to 0.5 again when $P_{\mathrm{ss}}=P_{\mathrm{eq}}$ (Fig. 9a3), since the weaker synapse from B to A is balanced by a phase that yields more response (more detail is explained in Sect. 3.2). For firing periods greater than $P_{\mathrm{eq}}$, the opposite relation holds, causing $\phi_{\mathrm{ss}}$ first to decrease to 0.41 and then increase back to 0.5. In contrast to $\phi_{\mathrm{ss}}$ varying between 0.41 and 0.58, $\phi_{\mathrm{st}}$ is always fixed at 0.5 due to identical neurons and synapses. Since the values of the plasticity profiles at the tails are less than the strength $\bar{g}=0.1$ of the static synapses, $P_{\mathrm{ss}}$ is slightly smaller than $P_{\mathrm{st}}$ for small (and large) intrinsic periods (Fig. 9a2). For a range of intermediate intrinsic periods, when the network synapses have plasticity, $P_{\mathrm{ss}}$ is almost equal to the network period with static synapses $P_{\mathrm{st}}$ (Fig. 9a2). The balancing effects of the two synaptic profiles ($g_{A}(P)$ being greater, $g_{B}(P)$ being smaller than $\bar{g}$ for $P_{\mathrm{ss}}<P_{\mathrm{eq}}$ and $g_{A}(P)$ being smaller, $g_{B}(P)$ being greater than $\bar{g}$ for $P_{\mathrm{ss}}<P_{\mathrm{eq}}$) causes $P_{\mathrm{ss}}$ and $P_{\mathrm{st}}$ to be almost equal for intermediate intrinsic periods. Thus, this choice of synaptic plasticity profiles provides the network the ability to produce a range of distinct phase relationships as the intrinsic period changes (Fig. 9a3). Note that the steady-state network period remains almost equal to its value as if no plasticity is included (Fig. 9a2). 

In the case of identical plasticity profiles, the neurons have the same preferred periods and the values of the plasticity profiles again approach 0.075 at the tails (Fig. 9b1). This causes $P_{\mathrm{ss}}$ to be smaller than $P_{\mathrm{st}}$ for small and large intrinsic periods (Fig. 9b2). For intermediate firing periods, the opposite holds. In contrast to the almost linear change in $P_{\mathrm{st}}$, $P_{\mathrm{ss}}$ changes nonlinearly as a function of the intrinsic periods. Also, in contrast to the nonlinear change in $P_{\mathrm{ss}}$, the phase of neuron A is fixed at 0.5, because both the neurons and their plasticity profiles are identical (Fig. 9b3). Hence, depending on the choice of plasticity profiles, the network coupled with synaptic plasticity can have the same period but different relative phases (Fig. 9a1–a3), or the same phases but different periods compared to the network coupled with static synapses (Fig. 9b1–b3).

#### 3.3.2 Conditions for Phase or Period Constancy

Short-term synaptic plasticity profiles are subject to change by neuromodulation and other long-term modifications [21]. In the previous section, we showed that as the synaptic plasticity profile changes, the network can maintain the network period or the relative activity phases among the network neurons. In this section, we examine the conditions on the steady-state synaptic plasticity profiles that would allow the network to maintain either a constant period or a constant phase. 

For this purpose, we make use of the fixed point equations for identical cells (3.26) obtained from the 2D map. The phase $\phi^{*}$ in (3.26) stand for the intrinsic phase of neuron A (3.2a). We use Eq. (3.12) and rewrite (3.26) as implicit functions of the steady-state phase of A $\tilde{\phi }$, network period *P* and synaptic preferred periods $P_{A}$ and $P_{B}$ as

$$\begin{array}{rl} F_{1}(P_{A},P_{B},\tilde{\phi },P) & =P-P_{0}\left[1-Z\left(\frac{P-\tilde{\phi }P}{P_{0}},g_{A}(P)\right)\right]\\ F_{2}(P_{A},P_{B},\tilde{\phi },P) & =P-P_{0}\left[1-Z\left(\frac{\tilde{\phi }P}{P_{0}},g_{B}(P)\right)\right] \end{array}$$

Let $F(P_{A},P_{B},\tilde{\phi },P)=(F_{1}(P_{A},P_{B},\tilde{\phi },P),F_{2}(P_{A},P_{B},\tilde{\phi },P))$. At the fixed point, $F(P_{A}^{*},P_{B}^{*},{\tilde{\phi }}^{*},P^{*})=(0,0)$. We would like to solve this equation for $P_{A}$ and $P_{B}$ as a function of *P* and $\tilde{\phi }$. Using the Implicit Function Theorem, the condition that needs to be satisfied is $\det(D_{P_{A},P_{B}}F)\neq 0$ at $\left(P_{A}^{*},P_{B}^{*},{\tilde{\phi }}^{*},P^{*}\right)$ where

(3.27) $$D_{P_{A},P_{B}}F{|}_{\left(P_{A}^{*},P_{B}^{*},{\tilde{\phi }}^{*},P^{*}\right)}={\left[\begin{array}{cc} \frac{\partial F_{1}}{\partial P_{A}} & \frac{\partial F_{1}}{\partial P_{B}}\\ \frac{\partial F_{2}}{\partial P_{A}} & \frac{\partial F_{2}}{\partial P_{B}} \end{array}\right]}_{\left(P_{A}^{*},P_{B}^{*},{\tilde{\phi }}^{*},P^{*}\right)}$$

The function $F_{1}$ does not depend on $P_{B}$, hence $\partial F_{1}/\partial P_{B}=0$. So, for the determinant to be nonzero, both $\partial F_{1}/\partial P_{A}$ and $\partial F_{2}/\partial P_{B}$ have to be nonzero. These terms are given as

$$\begin{array}{rl} \frac{\partial F_{1}}{\partial P_{A}}{|}_{\left(P_{A}^{*},P_{B}^{*},{\tilde{\phi }}^{*},P^{*}\right)} & =P_{0}\frac{\partial Z}{\partial y}\left(\frac{P^{*}(1-{\tilde{\phi }}^{*})}{P_{0}},g_{A}\left(P^{*}\right)\right)\frac{\partial g_{A}}{\partial P_{A}}\\ \frac{\partial F_{2}}{\partial P_{B}}{|}_{\left(P_{A}^{*},P_{B}^{*},{\tilde{\phi }}^{*},P^{*}\right)} & =P_{0}\frac{\partial Z}{\partial y}\left(\frac{{\tilde{\phi }}^{*}\cdot P^{*}}{P_{0}},g_{B}\left(P^{*}\right)\right)\frac{\partial g_{B}}{\partial P_{B}} \end{array}$$

One condition for the determinant to be nonzero is $\partial Z/\partial y(x,y){|}_{\left(P_{A}^{*},P_{B}^{*},{\tilde{\phi }}^{*},P^{*}\right)}\neq 0$; that is, the response of the neuron to perturbations should change with the change in the strength of the perturbation. This is a standard assumption on phase response curves with small perturbations. The other two conditions to be satisfied are $\partial g_{A}/\partial P_{A}{|}_{\left(P_{A}^{*},P_{B}^{*},{\tilde{\phi }}^{*},P^{*}\right)}\neq 0$ and $\partial g_{B}/\partial P_{B}{|}_{\left(P_{A}^{*},P_{B}^{*},{\tilde{\phi }}^{*},P^{*}\right)}\neq 0$, which, upon using Eq. (2.6), are equivalent to $P_{A}\neq P^{*}$ and $P_{B}\neq P^{*}$, respectively. In other words, the network period should be different from the synaptic preferred periods.

Under these three conditions, the Implicit Function Theorem guarantees that $P_{A}$ and $P_{B}$ can be expressed in terms of *ϕ* and *P* near $\left(P_{A}^{*},P_{B}^{*},{\tilde{\phi }}^{*},P^{*}\right)$. More precisely, there are neighborhoods *U* of $\left({\tilde{\phi }}^{*},P^{*}\right)$ and *W* of $\left(P_{A}^{*},P_{B}^{*}\right)$ such that, for each $(\tilde{\phi },P)\in U$, there exists a unique $(P_{A},P_{B})\in W$ such that $F(P_{A},P_{B},\tilde{\phi },P)=F(P_{A}(\tilde{\phi },P),P_{B}(\tilde{\phi },P),\tilde{\phi },P)=0$. Hence, there is a unique function $h=(h_{1},h_{2}):U\to W$ such that $F(h_{1}(\tilde{\phi },P),h_{2}(\tilde{\phi },P),\tilde{\phi },P)=0$ for every $(\tilde{\phi },P)\in U$.

We can interpret this result in two ways. First, around the fixed point $\left(P_{A}^{*},P_{B}^{*},{\tilde{\phi }}^{*},P^{*}\right)$, we can choose $\left({\tilde{\phi }}^{\prime },P^{*}\right)$ such that $P^{*}$ is fixed and ${\tilde{\phi }}^{\prime }\neq {\tilde{\phi }}^{*}$, for which there exist $\left(P_{A^{\prime }},P_{B^{\prime }}\right)$ that satisfy the fixed point equations (3.26). Hence, for a specific $P^{*}$, around a point with a phase ${\tilde{\phi }}^{\prime }$, there exist synaptic preferred periods $P_{A^{\prime }}$ and $P_{B^{\prime }}$ that enable the network to stay on the level set of $P^{*}$, while setting the phase equal to a new value ${\tilde{\phi }}^{\prime }$. In other words, it is possible to keep the network period constant and set the network phase to a new value by changing the synaptic plasticity profiles of the network neurons.

The second interpretation is that, around the fixed point $\left(P_{A}^{*},P_{B}^{*},{\tilde{\phi }}^{*},P^{*}\right)$, we can choose a $\left({\tilde{\phi }}^{*},P^{\prime }\right)$ such that ${\tilde{\phi }}^{*}$ is fixed and $P^{\prime }\neq P^{*}$, and can find $\left(P_{A^{\prime }},P_{B^{\prime }}\right)$ that satisfy the fixed point equations (3.26). This enables the network to stay on the level set for a specific ${\tilde{\phi }}^{*}$, while changing the network period to a new value $P^{\prime }$.

**Fig. 10:**
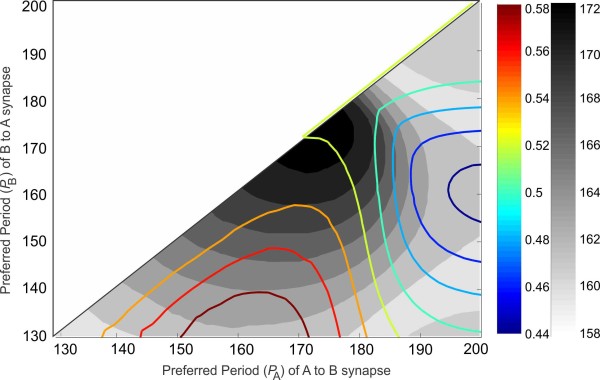
Period and phase locking for different steady-state synaptic plasticity profiles. The steady-state network period (*gray*) and phase (*colored*) are shown as a function of different steady-state synaptic plasticity profiles. *Colored curves* correspond to level sets of the phase. *The edges of the gray bands* correspond to the level sets of the network period. The plasticity profile of each synapse is marked by its preferred period

In the example demonstrated in Fig. 10, the intrinsic periods of the two neurons are kept constant but the two synaptic plasticity profiles are allowed to vary. As before, the synaptic plasticity profiles are changed only by shifting them along the period axis. We keep track of different synaptic plasticity profiles by the values of the synaptic preferred periods $P_{A}$ and $P_{B}$ (the peak of the profile). Figure 10 shows the changes in the network period and phase as the synaptic plasticity profiles of the neurons are varied. The neurons are identical with an intrinsic period $P_{0}$ of 137. The colored curves are subsets of the level sets of the phase; the phase of the network is fixed on a curve with a specific color. The gray bands correspond to the level sets of the network period. These level sets provide conditions for the network to maintain a specific period but have different phase relations, or vice versa, through varying the combination of synaptic preferred periods. 

#### 3.3.3 Networks of Non-identical Neurons

**Fig. 11:**
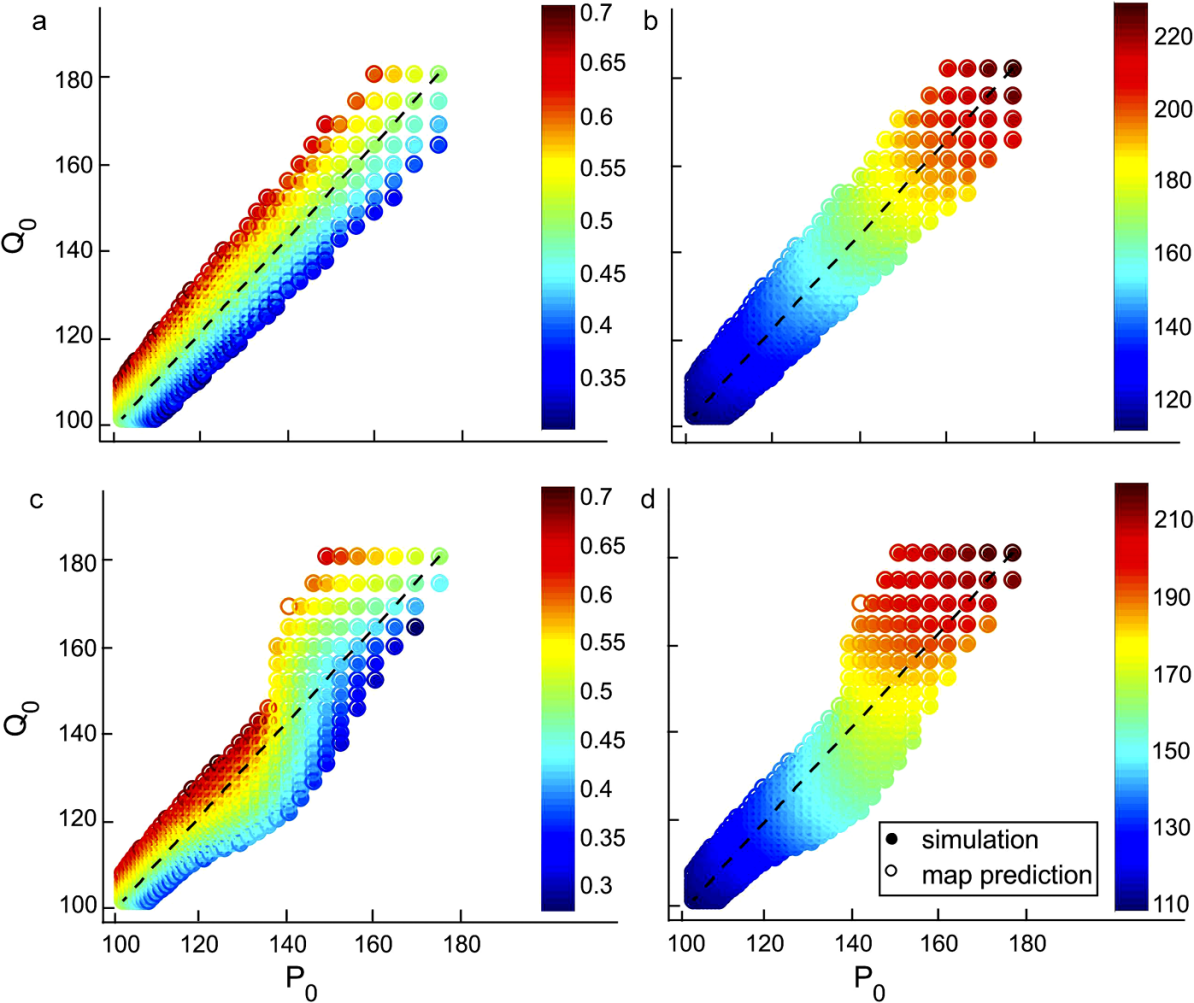
Coupling of non-identical M–L neurons. The phase of neuron A (**a** and **c**) and the period of the network (**b** and **d**) for coupled neurons with different intrinsic periods are shown for static synapses (**a** and **b**; $\bar{g}=0.1$) and when the network follows the synaptic plasticity profile (**c** and **d**; $P_{A}=150$, $P_{B}=190$). The axes are the intrinsic periods of the two neurons. Plasticity adds nonlinearity to the period and phase distribution. *Filled circles* denote simulation results whereas *open circles* denote the map predictions. The map yields predictions very close to the simulations in most cases

We now examine a network of two non-identical M–L neurons. The neurons are chosen to have different intrinsic periods by applying different levels of external current but otherwise using the same parameters. We consider the two cases where the synapses are static or they follow steady-state synaptic plasticity profiles and compare the predictions of the 1D map (3.7) and the 2D map (3.24) with the simulations of the corresponding model equations. We let the preferred period of the A to B synapse be $P_{A}=150$ and from neuron B to A be $P_{B}=190$ for the case with synaptic plasticity. The results are shown in Fig. 11. 

Note that the maps continue to give good predictions when the neurons are not necessarily identical. The difference between the simulations (filled circles) and the map predictions (open circles) is indistinguishable in most cases. The diagonal corresponds to coupling of identical neurons. Moving away from the diagonal, the difference between the intrinsic periods of the neurons increases and eventually prevents the neurons to phase lock in a 1:1 manner because the fixed point equation (3.8) is not satisfied anymore. These are the limits of the region shown in Fig. 11. Observe that the limits determined by the map and the simulations overlap except at one single case shown only by an open circle in Figs. 11c and 11d. Here, the map predicts that a 1:1 solution exists, while the simulation does not converge to that. In this case, the simulation shows that the firing order between the neurons is not preserved which violates the 1:1 firing assumption of the map.

The phase of neuron A equals 0.5 on the diagonal in the static coupling case (Fig. 11a). It decreases (resp. increases) linearly as $Q_{0}$ moves down (resp. up) from the diagonal. This behavior can be predicted by analyzing Eq. (3.8). In the identical network, where $P_{0}=Q_{0}$, the activity phases (${\tilde{\phi }}^{*}={\tilde{\theta }}^{*}=0.5$), and the intrinsic phases ($\phi^{*}=\theta^{*}=0.598$) of the two neurons are equal and hence $Z_{A}(\phi^{*})=Z_{B}(\theta^{*})$. If the solution is perturbed such that $P_{0}>Q_{0}$, then the response of neuron A to synaptic inputs from neuron B must be smaller than the response of neuron B for the Eq. (3.8) to be satisfied. The PRC of the neurons has a negative slope at this intrinsic phase $\phi^{*}$ (Fig. 1b). So, the intrinsic phase *ϕ* of neuron A in the perturbed solution must be smaller than $\phi^{*}$ for $Z_{A}(\phi )$ to be smaller than $Z_{A}(\phi^{*})$. As the function (3.12) relating *ϕ* and $\tilde{\phi }$ is monotone increasing, the activity phase $\tilde{\phi }$ of neuron A in the perturbed solution must also be smaller than ${\tilde{\phi }}^{*}$. Hence, as the difference $P_{0}-Q_{0}$ increases (resp. decreases), the phase of neuron A decreases (resp. increases). The period of the network increases linearly as the intrinsic periods increase in the static coupling case (Fig. 11b). Due to symmetry in the synaptic strengths, the distribution of the period is symmetric with respect to the diagonal.

When the synapses are plastic, some 1:1 phase-locked solutions that existed with static coupling no longer exist, while new solutions may emerge (Figs. 11c and 11d). Due to asymmetry in the synaptic plasticity profiles, the upper bound for the difference in intrinsic periods that allow a 1:1 phase-locked solution varies. This can be seen by comparing the circles in the top row and rightmost column of Figs. 11c and 11d. At the right top corner, $P_{0}=Q_{0}=181$, and the network has an anti-phase solution. If $Q_{0}$ is fixed, while $P_{0}$ decreases, the network continues to phase lock in a 1:1 solution for $P_{0}\geq 152.1$. On the other hand, if $P_{0}$ is fixed, while $Q_{0}$ decreases, then the network phase locks in a 1:1 solution only when $Q_{0}\geq 174.8$. Although the absolute difference between the intrinsic periods are equal, different plasticity profiles causes convergence in one case but not the other. This can be understood by considering (3.25). For the identical cell case where $P_{0}=Q_{0}=181$, the network period is equal to $P^{*}=219.5$. Due to the selection of the plasticity profiles, $g_{A}(P^{*})<g_{B}(P^{*})$, since $P^{*}$ is close to $P_{B}=190$ than it is to $P_{A}=150$. As a result, neuron A receives stronger synaptic input from neuron B at the steady state (as $g_{B}(P^{*})$ determines $g_{B\to A}$). The firing periods of both neurons must be equal at the fixed point. This is only possible if neuron B receives synaptic input at a phase that yields a larger response than that of neuron A. Hence, although the neurons are identical, the difference in their plasticity profiles causes a phase-locking solution different from anti-phase. Assume now that $Q_{0}>P_{0}$. Then the relation $g_{A}(P)<g_{B}(P)$ will still hold as *P* will stay close to $P^{*}$. In this case, the synaptic strength received by neuron A will be larger, while its intrinsic period will be smaller than that of B. These two opposing effects will let the network continue having a solution until the difference between the intrinsic periods are too large to be compensated for and (3.25) are not satisfied. On the other hand, if the symmetric solution is perturbed such that $P_{0}>Q_{0}$, then the synaptic strength received by neuron A and its intrinsic period will both be larger than those of neuron B. The phase of neuron B must increase further and yield a larger response to compensate for these adding effects. But when the PRC reaches a maximum in absolute value and starts to decrease, there would be no phase value that would compensate for these effects and the network will not be able to have a 1:1 solution. This explains why the limits of the regions in the case with synaptic plasticity are not symmetrical.

In general, whether (3.25) are satisfied or not depends on the intrinsic periods $P_{0}$, $Q_{0}$ and the values of the PRCs as in the static map case. But in this case the values of the PRCs are also determined by two factors, the phase of inhibition received, and its strength—which is determined by the network period. Hence, the phase of neuron A is a determined both by the interaction of intrinsic periods and the plasticity profiles. This is also responsible for the nonlinearity in the distribution of phase. The level curves of phase are nonlinear in the case with synaptic plasticity as opposed to the linear level curves in the static coupling case.

## 4 Discussion

In the analysis of an oscillatory network, the steady-state activity of the network can often be reduced to the study of a return map. The advantage of using maps is that it often allows the network dynamics to be understood by tracking empirically observable characteristics such as period and phase. Here, we derive such a map for a two-cell network coupled with inhibitory synapses with the goal of understanding how short-term synaptic plasticity and other factors determine the network period and the relative activity phase of the two neurons. Our results show that the information on the network period and phase can be obtained using maps that keep track of observable network variables such as the intrinsic periods of the neurons involved, their phase response curves and the synaptic plasticity profiles: relationships describing how the synaptic strength depends on input frequency. These variables can be readily determined experimentally with “feed-forward” measurements where the input is controlled by the experimenter and the output is measured. For example, the strength of a synapse can be measured at all frequencies simply by driving the presynaptic neuron at different rates and measuring the postsynaptic current. In fact, the current study was motivated by our experimental measurements of these types of network variables in the crab stomatogastric pyloric network [22-24]. 

There are several prior works that utilize PRCs and map-based techniques to understand phase locking [1-13]. Of particular interest is the result of Cui et al. [5] who use a functional PRC (fPRC) that is calculated from actual experimental measurements of Aplysia pacemaker neurons. Cui et al. show that the fPRC differs from the single pulse PRC (as was used in this paper) due to accommodation of the pacemaker neurons. They then go on to use the fPRC to study phase-locking in a coupled network by deriving a map that encodes how a neuron responds to a period input that arrives a fixed time after the firing of the cell. By linearizing about a fixed point of their 1D map, they find conditions for the existence and stability of 1:1 phase-locked solutions. Their predictions from the fPRC method are better matched to simulations than predictions from a conventional single-pulse PRC. Importantly, their fPRC methods do not depend on the exact shape of the PRC but rather on the effect on the cycle period based on the time the input was given. This is a statistic that is easily found in experiments. Moreover, their results are obtained from combining feed-forward processes as opposed to directly studying a feedback map, which they call open-looped versus closed-looped.

Our results complement those of Cui et al. in the sense that we relate cycle-to-cycle changes in the period independent of how those changes arise, allowing us to also use experimentally obtainable information to derive the maps. Our maps are also based on assumptions that are consistent with Cui et al.’s assumption that the closed-loop behavior of a system can be predicted by knowing the open-looped behavior of some of its components. Our results extend those of Cui et al. and other prior works in that we allow the timing of inputs to vary on a cycle by cycle basis that is determined by the synaptic plasticity profile of the presynaptic neuron. This results in a higher-dimensional map arising by specifically considering the dynamics of synaptic facilitation and depression on a cycle by cycle basis. This yields a 3D map when plasticity is present only in one direction of the two-cell network, or a 5D map if present in both directions. When we used the steady-state synaptic plasticity profile, both cases reduce to a 2D map. For this 2D map, we derived a geometric method that generalizes cobwebbing in a 1D map to allow us to study the existence and stability of fixed points. For a generic 1D map, Π(x), the intersection of the curve y=Π(x) with the curve y=x, and the slope at that point, determine existence and stability of the fixed point. In our generalized 2D case, given maps $\Pi_{1}(x,y)$ and $\Pi_{2}(x,y)$, it is the intersection of these surfaces with appropriate planes that yield two curves. It is the intersection of the projection of these two curves onto a common plane that determines existence of the fixed point. Stability is more complicated than just checking the slopes at the point of intersection. We showed how it could depend on both the PRC and the synaptic plasticity profile.

In this study, we considered a general form of short-term synaptic plasticity which is a combination of short-term facilitation and depression. We modeled such a synapse using an ad hoc model as described previously [16]. The advantage of this model is that the extent to which facilitation or depression is a dominant factor can be simply determined by changing the model parameters. Our analysis progressed through a network of two neurons with static synapses, the same network but with one synapse having plasticity and finally with both synapses showing plasticity. The analysis of a two-cell network with static synapses yields a 1D map [6,8]. Including synaptic plasticity increases the dimension of the map because variables underlying synaptic dynamics must be tracked as well. The change in synaptic strength due to the plasticity means that the PRCs of the neurons also change. Our analysis shows that these higher-dimensional maps can accurately predict the steady-state phase and period of the network, as seen in comparisons with numerical simulations of the underlying ODEs. 

In experimental measurements, synaptic plasticity profiles are often measured using repetitive input pulses or waveforms and reported at steady state, i.e., the steady-state strength of the synapse is known for each stimulation frequency [23,25,26]. In most cases, the mechanisms that underlie these synaptic dynamics are unknown and it is therefore impossible to track how synaptic strength changes as a function of frequency on a cycle-to-cycle basis. One of the interesting findings from our work is that the prediction of the higher-dimensional map obtained when using dynamics of the synapse is the same as a lower-dimensional map that uses only the steady-state plasticity profile. In other words, the network output is dependent on the steady-state strength independent of the mechanisms through which this synaptic strength is actually generated. In turn, this allows an experimentalist to understand the effects of, say a synaptic neuromodulator, on the network output simply by understanding the effect on a single component such as the synaptic plasticity profile. 

The results of our maps help us understand the role of synaptic dynamics in determining the relative phase between two neurons in an oscillatory network. For example, neurons in the crustacean pyloric oscillatory network, involve multiple reciprocally inhibitory connections. Pyloric oscillations are quite stable in individual preparations and are generated by a pacemaker group of neurons (AB/PD) which make reciprocally inhibitory connections with a single follower neuron, LP. The analysis of this reciprocally inhibitory network provided the motivation for the current study. As in other rhythmic motor networks, the pyloric network neurons maintain a constant phase relationship even when these phases are measured in different animals [27]. Surprisingly this tight phase relationship is maintained despite a large variability in the pyloric cycle period (1–2.5 s) across preparations. In fact, different preparations differ both in the intrinsic periods of the neurons involved as well as the synaptic plasticity profiles. The results of the current study indicate that the pyloric network could maintain constant phase relationships, even in different animals, by tuning the synaptic plasticity profiles along the level sets of phase (Fig. 10). Alternatively, if the relative activity phases of the neurons involved in producing the network oscillations are not an essential component of the network output, but the network must maintain a constant period, the maps we have derived can be used to establish the relationships that could produce a constant frequency output. These are plausible strategies for all rhythmic motor networks in which the output is tightly constrained by the proper phase of muscle movements to produce meaningful behavior.

An interesting implication of our results is that if the network period coincides with the synaptic preferred periods, it is not possible to uniquely prescribe the synaptic profiles in terms of the network period and the relative phase of the neurons (Eq. (3.27)). If the level sets of phase, described in Fig. 10, provide a unique rule for the network to tune its synaptic plasticity profiles for phase maintenance, then the network period should avoid the synaptic preferred periods. Additionally, by avoiding the periods at which the synaptic strengths are maximal, the network can operate with a larger degree of flexibility and perhaps more efficiently. This is in fact the case for the synapses between the AB/PD pacemaker neurons and the follower LP neuron in the crustacean pyloric network. The network period is around 1–2.5 s, in a range of values that is larger than the preferred periods of the synapses (∼0.5 Hz) [23]. Hence, our findings give an insight for this experimentally observed fact. 

In conclusion, we have shown that the frequency-dependent information on synapses can be combined with the PRCs of oscillatory neurons to predict the activity period and phases of a coupled network using maps derived from empirically observable relationships. It is plausible that a similar approach can be used whenever there is frequency-dependent information about the network components to construct maps that predict the activity of an oscillatory network, even when the synapses include excitatory connections or obey different plasticity profiles. In relationship to the crustacean pyloric network that motivated this study, current experimental work in our lab involves measuring the changes in the synaptic plasticity profiles and the neuronal PRCs in the presence of different neuromodulators to see whether the maps derived here can predict how the network output changes in the presence of these modulators.

## Competing Interests

The authors declare that they have no competing interests.

## Authors’ Contributions

All authors contributed equally to the writing of this paper. All authors read and approved the final manuscript.

## References

[B1] Wang S Chandrasekaran L Fernandez FR White JA Canavier CC Short conduction delays cause inhibition rather than excitation to favor synchrony in hybrid neuronal networks of the entorhinal cortex PLoS Comput Biol 2012 4 1 Article ID e1002306 10.1371/journal.pcbi.1002306 PMC3252263 22241969

[B2] Sieling FH Archila S Hooper R Canavier CC Prinz AA Phase response theory extended to nonoscillatory network components Phys Rev E, Stat Nonlinear Soft Matter Phys 2012 4 5-2 Article ID 056208 10.1103/PhysRevE.85.056208 PMC3501682 23004844

[B3] Oprisan SA Prinz AA Canavier CC Phase resetting and phase locking in hybrid circuits of one model and one biological neuron Biophys J 2004 4 4 2283 2298 10.1529/biophysj.104.046193 15454430 PMC1304653

[B4] Maran SK Canavier CC Using phase resetting to predict 1:1 and 2:2 locking in two neuron networks in which firing order is not always preserved J Comput Neurosci 2008 4 1 37 55 10.1007/s10827-007-0040-z 17577651 PMC2719962

[B5] Cui J Canavier CC Butera RJ Functional phase response curves: a method for understanding synchronization of adapting neurons J Neurophysiol 2009 4 1 387 398 10.1152/jn.00037.2009 19420126 PMC2712257

[B6] Huang X: **Using feed-forward networks to infer the activity of feedback neuronal networks**. *PhD thesis*. New Jersey Institute of Technology; 2011.

[B7] Netoff TI Banks MI Dorval AD Acker CD Haas JS Kopell N White JA Synchronization in hybrid neuronal networks of the hippocampal formation J Neurophysiol 2005 4 3 1197 1208 15525802 10.1152/jn.00982.2004

[B8] Dror RO Canavier CC Butera RJ Clark JW Byrne JH A mathematical criterion based on phase response curves for stability in a ring of coupled oscillators Biol Cybern 1999 4 1 11 23 10.1007/s004220050501 20809292

[B9] Achuthan S Canavier CC Phase-resetting curves determine synchronization, phase locking, and clustering in networks of neural oscillators J Neurosci 2009 4 16 5218 5233 10.1523/JNEUROSCI.0426-09.2009 19386918 PMC2765798

[B10] Oprisan SA Existence and stability criteria for phase-locked modes in ring neural networks based on the spike time resetting curve method J Theor Biol 2010 4 2 232 244 10.1016/j.jtbi.2009.09.036 19818355

[B11] Ermentrout GB n:m phase-locking of weakly coupled oscillators J Math Biol 1981 4 327 342 10.1007/BF00276920

[B12] Pervouchine DD Netoff TI Rotstein HG White JA Cunningham MO Whittington MA Kopell NJ Low-dimensional maps encoding dynamics in entorhinal cortex and hippocampus Neural Comput 2006 4 11 2617 2650 10.1162/neco.2006.18.11.2617 16999573

[B13] Woodman MM Canavier CC Effects of conduction delays on the existence and stability of one to one phase locking between two pulse-coupled oscillators J Comput Neurosci 2011 4 2 401 418 10.1007/s10827-011-0315-2 21344300 PMC3130804

[B14] Abbott LF Regehr WG Synaptic computation Nature 2004 4 7010 796 803 10.1038/nature03010 15483601

[B15] Izhikevich EM Desai NS Walcott EC Hoppensteadt FC Bursts as a unit of neural information: selective communication via resonance Trends Neurosci 2003 4 3 161 167 10.1016/S0166-2236(03)00034-1 12591219

[B16] Markram H Wang Y Tsodyks M Differential signaling via the same axon of neocortical pyramidal neurons Proc Natl Acad Sci USA 1998 4 9 5323 5328 10.1073/pnas.95.9.5323 9560274 PMC20259

[B17] Morris C Lecar H Voltage oscillations in the barnacle giant muscle fiber Biophys J 1981 4 1 193 213 10.1016/S0006-3495(81)84782-0 7260316 PMC1327511

[B18] Ermentrout B Terman DH Mathematical Foundations of Neuroscience 2010 Springer, New York

[B19] Drover JD Tohidi V Bose A Nadim F Combining synaptic and cellular resonance in a feed-forward neuronal network Neurocomputing 2007 4 10-12 2041 2045 10.1016/j.neucom.2006.10.135 19079739 PMC2600776

[B20] Ermentrout B Simulating, Analyzing, and Animating Dynamical Systems 2002 Society for Industrial and Applied Mathematics, Philadelphia

[B21] Johnson BR Brown JM Kvarta MD Lu JY Schneider LR Nadim F Harris-Warrick RM Differential modulation of synaptic strength and timing regulate synaptic efficacy in a motor network J Neurophysiol 2011 4 1 293 304 10.1152/jn.00809.2010 21047938 PMC3023374

[B22] Nadim F Zhao S Zhou L Bose A Inhibitory feedback promotes stability in an oscillatory network J Neural Eng 2011 4 Article ID 065001 10.1088/1741-2560/8/6/065001 PMC3407465 22058272

[B23] Tseng HA Nadim F The frequency-dependent response of synapse in an oscillatory network Soc Neurosci Abstr 2010 4 Article ID 489.12

[B24] Nadim F Zhao S Bose A Butera RJ, Prinz A, Schultheiss NW A PRC description of how inhibitory feedback promotes oscillation stability Phase Response Curves in Neuroscience 2012 Springer, Berlin 399 418

[B25] Fortune ES Rose GJ Short-term synaptic plasticity contributes to the temporal filtering of electrosensory information J Neurosci 2000 4 18 7122 7130 10995860 10.1523/JNEUROSCI.20-18-07122.2000 PMC6772817

[B26] Feng L Molnar P Nadler JV Short-term frequency-dependent plasticity at recurrent mossy fiber synapses of the epileptic brain J Neurosci 2003 4 12 5381 5390 12832564 10.1523/JNEUROSCI.23-12-05381.2003 PMC6741152

[B27] Bucher D Prinz AA Marder E Animal-to-animal variability in motor pattern production in adults and during growth J Neurosci 2005 4 7 1611 1619 10.1523/JNEUROSCI.3679-04.2005 15716396 PMC6725924

